# Guideline-Incorporated Large Language Model-Driven Evaluation of Medical Records Using MedCheckLLM

**DOI:** 10.2196/53335

**Published:** 2025-04-24

**Authors:** Marc Cicero Schubert, Stella Soyka, Wolfgang Wick, Varun Venkataramani

**Affiliations:** 1 Department of Neurology, University Hospital Heidelberg, Im Neuenheimer Feld 400, Heidelberg, 69120, Germany, 49 6221548630

**Keywords:** large language models, AI, electron medical records, checklists, LLM, language model, NLP, natural language processing, records, documentation, documents, framework, conceptual, machine learning, artificial intelligence, evidence, evaluate, evaluation, guideline, health care

## Abstract

The study introduces MedCheckLLM, a large language model–driven framework that enhances medical record evaluation through a guideline-in-the-loop approach by integrating evidence-based guidelines.

## Introduction

Large language models (LLMs) have demonstrated enormous potential in assessing complex datasets in health care across many applications [1,2]. One underexplored area is their application for the reliable evaluation of medical documents. The automated evaluation of these documents has the potential to enhance patient safety. The system’s reasoning process must be (1) transparent and comprehensible to human evaluators and (2) guided by established medical guidelines proven to increase patient safety [3].

In this study, we introduce a framework that consists of a multistep approach for medical record evaluation that incorporates guidelines into the evaluation process (ie, guideline-in-the-loop). Our proposed algorithm, MedCheckLLM, is an LLM-driven, structured reasoning mechanism designed to automate the evaluation of medical records against evidence-based guidelines. The guidelines are deterministically accessed and returned to the LLM as input without further model fine-tuning. This strict separation of LLM and guidelines is expected to increase the validity and interpretability of the evaluations. The approach's step-by-step structure could improve transparency in clinical applications. The primary objective of this research is to introduce the conceptual framework and assess its feasibility.

## Methods

**Figure 1.**
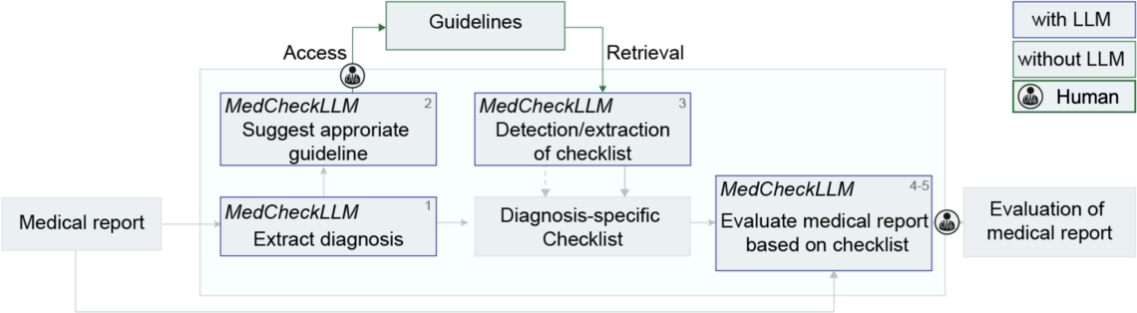
Workflow of MedCheckLLM. A medical report including medical history, diagnosis and treatment is provided as input. First, the LLM identifies the given diagnosis. Second, it suggests a medical guideline for evaluation of the medical report, with a human medical expert making the final selection. Then, independently of the LLM, the selected guideline is accessed, and diagnosis-specific text is extracted and inputted into the LLM. Subsequently, the LLM determines whether the input guideline text is in checklist format; if not, it extracts a checklist. Using this diagnosis-specific checklist, the LLM evaluates the medical report based on the diagnosis-specific checklist. Finally, a human expert assesses the LLM evaluation. Dashed arrow: Checklist extraction instead of detection of checklist format. Blue box: Component uses an LLM. Green Box: Components do not use an LLM.

The MedCheckLLM algorithm begins by extracting a patient’s diagnosis from the medical report (Figure 1). Based on the diagnosis, it suggests an appropriate guideline. A human medical expert makes the final guideline selection. Guidelines are then accessed independently of the LLM’s mechanisms using programmatically built interfaces for guideline retrieval. Subsequently, guidelines are provided as input to the LLM and are either identified as already formatted in a usable checklist or converted into a checklist. This diagnosis-specific checklist is used to assess the medical report by the LLM, with a final verification by a human medical expert. To test this approach, we used expert-validated simulated medical reports (simulated dataset) and physician-generated medical reports (physician dataset). Performance was analyzed for patient histories with headaches using guidelines from the International Headache Society and the physician dataset for four further neurological diagnoses (ie, border zone infarction, meningitis, neuromyelitis optica, and subarachnoid haemorrhage). The validity of this method was further analyzed by evaluating generated doctor’s notes with a correct diagnosis compared to doctor’s notes with a false diagnosis. The LLMs, GPT-4 and Claude-3 were used for testing (see Multimedia Appendix 1).

## Results

We evaluated the medical report analysis conducted by MedCheckLLM for various headache diagnoses. In the simulated dataset, MedCheckLLM (based on GPT-4 and Claude-3, Table 1) extracted the specified diagnosis correctly in 100% of cases from a list of 61 possible diagnoses from The International Classification of Headache Disorders-3 [4]. The model suggested existing evidence-based guidelines in 70.59% (12/17) of medical reports and detected the format of the guidelines as checklists in 100% of the cases (N=17). MedCheckLLM accurately evaluated 87% (67/ 77) of checklist items. Performance on the physician dataset showed an accurate evaluation in 77.4% (24/ 31) of checklist items (Table 2). It identified incorrect diagnoses where the stated diagnosis did not align with the content of the doctor’s letters in 94.1% (16/17) of cases, while it correctly recognized 100% (N=17) of letters with matching diagnoses.

**Table 1.** Performance of MedCheckLLM on the simulated dataset.

Elements of algorithmic structure	GPT-4 performance, % (n/N)	Claude-3 performance, % (n/N)	Explanation of specific task of each element
Extracting stated diagnosis	100 (17/17)	100 (17/17)	Extract the diagnosis that is stated in the medical report
Suggestion of existing guidelines	70.6 (12/17)	58.8 (10/17)	Suggest a guideline that should be used to evaluate the medical report
Detection of checklist	100 (17/17)	100 (17/17)	Detect whether the accessed guidelines are in a structured checklist- criteria format
Evaluation of diagnostic criteria (checklist items)	87 (67/77)	83.8 (62/74)	Assess whether the criteria listed in the checklist are met in the medical report
Evaluation of letters with correct diagnosis (clinical descriptions and diagnosis match)	100 (17/17)	94.1 (16/17)	Assess whether the diagnosis stated in the medical report aligns with the clinical descriptions
Evaluation of letters with false diagnosis (clinical descriptions and diagnosis do not match)	91.4 (16/17)	91.4 (16/17)	Evaluate whether the diagnosis that is stated in the medical report fits the clinical descriptions

**Table 2.** Performance of MedCheckLLM on the physician dataset.

Element of algorithmic structure	Stroke	Meningitis	Neuromyelitis optica	Subarachnoid hemorrhage
Extracting stated diagnosis	Yes	Yes	Yes	Yes
Suggestion of existing guidelines^a^	Yes, applicable	Yes, partially applicable	Yes, applicable	Yes, partially applicable
Creation of checklist, level of detail^a^	Yes, moderate detail	Yes, moderate detail	Yes, thorough detail	Yes, minimal detail
Evaluation of diagnostic criteria, % (n/N)	100 (7/7)	66.7 (4/6)	87.5 (7/8)	60 (6/10)

a The responses were classified as yes, and partially applicable,applicable, or minimal, moderate, or thorough detail.

## Discussion

The framework of MedCheckLLM represents a promising approach for a comprehensive, guideline-anchored review of electronic health records. It holds the potential to function as a quality assurance framework throughout patient care due to its advantages of separate partitioning of the LLM and the guidelines, rather than training guidelines into an LLM. The flexibility of this approach allows for immediate implementation of guideline updates or the option to implement customized protocols for subgroups of patients. Due to the checklist-based approach, each item can be verified individually, thus increasing the algorithm’s interpretability, which is crucial in health care settings [5]. Due to the LLM’s subpar guideline suggestion capability, medical experts are integrated at this step to ensure that established guidelines are used. Further research is essential to advance the development of LLM-driven methods for extracting checklists from unstructured guidelines, as well-structured guidelines are crucial for detailed, high-quality checklists. Further, this framework facilitates improved data mining practices in electronic health records [6]. In the future, it is crucial to address privacy concerns to ensure the ethical application of these powerful tools in real-world clinical settings [7-9].

## Supplementary material

10.2196/53335 Multimedia Appendix 1 Large language models used in this study.

## Abbreviations

LLM: large language model
