# Supplementary material for: Guideline-Incorporated Large Language Model-Driven Evaluation of Medical Records Using MedCheckLLM
Source: JMIR Form Res. 2025 Apr 24;9:e53335. doi: 10.2196/53335 (PMC12045122; doi:10.2196/53335)
Supplement: Multimedia Appendix 1 [file formative-v9-e53335-s001.pdf]

Supplement 1

Contents

Supplementary Methods 1
Large Language Models used in this study 1
Simulated Medical Reports 1
MedCheckLLM Components 1
Diagnostic Criteria – Example 2
Physician Dataset 2
Ethical Considerations 2
Prompts Used in the Study 3
Simulated Dataset 3
Physician Dataset 4
Supplementary References 5

Supplementary Methods

The study was conducted between July 24th 2023 and September 16th 2024.

Large Language Models used in this study

Open-AI model used in this study: model="gpt-4-0613". The model was accessed via the Open-AI API for the simulated headache dataset and using the GPT-4 web interface for the physician dataset. Anthropic model used in this study: model= "claude-3-sonnet-20240229". The model was accessed via the Claude API. MedCheckLLM was evaluated based on both models separately.

Simulated Medical Reports

The simulated medical reports used in this study included patients' medical history, examination outcomes, diagnosis, and treatment strategies and can be accessed on https://github.com/venkataramani-lab/MedCheckLLM. Only GPT-4 was used for creating the simulated medical reports.

MedCheckLLM Components

Module	Task	Input	Output
1. Extracting stated diagnosis	identifying the diagnosis that is stated in the medical report	Medical report, dictionary of diagnoses	Extracted diagnosis
2. Suggestion of existing guidelines	Suggest appropriate guidelines based on the medical report	Medical report	Suggested guideline
3. Detection of checklist	Assess whether guideline is in structured checklist format, or return output in checklist format	Guideline text	Assessment about checklist format, or checklist

4. Evaluation of diagnostic criteria	Evaluate whether checklist items are met in the medical report or not	Medical report, Diagnosis, Guideline checklist	List of evaluated checklist items
5. Evaluation of congruence of clinical descriptions and diagnosis	Evaluate whether the diagnosis that is stated in the medical report fits to the clinical descriptions	Medical report	Assessment whether the diagnosis and clinical descriptions fit together

The first module has the task of identifying the diagnosis that is stated in the medical report. It needs as input the medical report and a dictionary of diagnoses and returns the extracted diagnosis. The second module has the task of suggesting appropriate guidelines based on the medical report. It needs as input the medical report and returns a suggestion for a guideline for this letter. The third module assesses whether the retrieved guidelines are in a structured format that can be used as checklist. It has as input the accessed guideline portion and returns whether the output is in a checklist format or returns it as a checklist if not. The fourth model evaluates whether the medical report fulfils the diagnostic criteria from the guideline checklist. It has as input the medical report, the diagnosis and the guideline-based checklist, and returns a list of checklist items, with an evaluation of each checklist item and a comment. The fifth module evaluates whether the diagnosis that is stated in the medical report fits to the clinical descriptions. It takes as input the medical report, and returns the assessment of whether or not the diagnosis fits the clinical descriptions.

Diagnostic Criteria – Example

International Classification of Headache Disorders (ICHD-3) of the International Headache Society.[1]

Hypnic headache

A. Recurrent headache attacks fulfilling criteria B-E
B. Developing only during sleep, and causing wakening
C. Occurring on ≥10 days/month for >3 months
D. Lasting from 15 minutes up to 4 hours after waking
E. No cranial autonomic symptoms or restlessness
F. Not better accounted for by another ICHD-3 diagnosis

The International Classification of Headache Disorders 3rd edition can be accessed via https://ichd-3.org/.

Physician Dataset

The letters for the physician dataset were created by two physicians based on realistic clinical scenarios. Subsequently, they were translated using GPT-4. Evaluation of the physician dataset letters was performed using the web interface of GPT-4. Each step of the framework was evaluated separately. The guidelines used for each disorder [2-5] were given as input either as plain text or uploaded as pdf file. Output was saved and analyzed. Checklists were assessed solely based on their level of detail and scored as either minimal detail (vague general instructions), moderate detail (partially broad and partially specific), thorough detail (specifies precise criteria). Checklist evaluation was performed by assessing whether the LLM evaluated each checklist item.

Ethical Considerations

This study was exempt from institutional review board approval as it did not involve human participants. Therefore, informed consent, privacy protections, compensation details, and participant identification measures were not applicable to this study.

Prompts Used in the Study

Simulated Dataset

Create Example Medical Report

{"role": "user", "content": "Please write an example medical report for a person with the diagnosis " + diagnosis + ".\n" }

Extract Diagnosis

{"role": "user", "content": "You are supposed to extract a diagnosis of a medical report. Of the choices, return the approriate headache type key for the diagnosis in the following medical report:\n\nKeys:" + KEYS +"\nMedical Report:\n" + report + "\n\n Please return ONLY the key, so that I can access the dictionary directly., e.g.:\n14.1 Headache not elsewhere classified"}

Select Guideline

{"role": "user", "content": '''Please examine the following medical report and provide:

- The leading symptom

- potential syndrome

- The diagnosis

- The name of a relevant, established medical guideline for the leading symptom.

\n Do not return actual medical advice but the name of a relevant medical guideline. \nMedical Report:\n''' + report + '''\n\n'''}

Turn into Checklist

{"role": "user", "content": f''' I will provide you with part of a medical guideline. If it is in a format that could be used as a checklist, return the word 'CHECKLIST'. Only return the word 'CHECKLIST', not the actual checklist itself.

If it is a continuous, long text with complete sentences, try to extract a checklist from the guideline and return your checklist.

\n

Guideline:

\n {guideline_out}

'''}

Evaluate Letter

{"role": "user", "content": """

I will provide you with a checklist guideline and a medical report. Please assess the doctor's letter based on the checklist and consider the following: Checklist items are usually numbered A,B,C,D,E ... Understand what is meant by for example: Any headache fulfilling criterion B and C. Then assess the letter and return your results:

- For each checklist item separatedly:

- Whether the checklist item was thoroughly addressed in the doctor's letter (0-5: not covered at all (0) - comprehensively covered (5)).

- Comment.

- Additional comments. '''}

Correct Diagnosis

{"role": "user", "content": """

I will provide you with a medical report. Please assess whether the doctor's letter identified the correct diagnosis. Return the following results:

- The diagnosis stated in the doctor's letter

- The diagnosis that you believe the patient actually has

- whether the stated and actual diagnosis are the same (yes/no)

- Additional comments. """},

{"role": "user", "content": "Letter:\n" + letter}

Physician Dataset

Extract Diagnosis

"You are supposed to extract a diagnosis of a medical report. Medical report: {medical_report}"

Select Guideline

''Please examine the following medical report and provide:

- The leading symptom

- potential syndrome

- The diagnosis

- The name of a relevant, established medical guideline for the leading symptom.

Do not return actual medical advice but the name of a relevant medical guideline.

Medical Report: {medical_report}”

Turn into Checklist

“I will provide you with part of a medical guideline. If it is in a format that could be used as a checklist, return the word 'CHECKLIST'. If it is a continuous, long text with complete sentences, try to extract a checklist from the guideline and return your checklist.

Guideline: {guideline_text/guideline_pdf}"

Evaluate Letter

“I will provide you with a checklist guideline and a medical report. Please assess the doctor's letter based on the checklist and consider the following: Checklist items are usually numbered A,B,C,D,E ... Understand what is meant by for example: Any headache fulfilling criterion B and C. Then assess the letter and return your results:

- For each checklist item separatedly:

- Whether the checklist item was thoroughly addressed in the doctor's letter (0-5: not covered at all (0) - comprehensively covered (5)).

- Comment.

- Additional comments.

Checklist: {checklist}

Medical report: {medical_report}'"

Supplementary References

1. *Headache Classification Committee of the International Headache Society (IHS) The International Classification of Headache Disorders, 3rd edition.* Cephalalgia, 2018. **38**(1): p. 1-211.
2. Powers, W.J., et al., *Guidelines for the Early Management of Patients With Acute Ischemic Stroke: 2019 Update to the 2018 Guidelines for the Early Management of Acute Ischemic Stroke: A Guideline for Healthcare Professionals From the American Heart Association/American Stroke Association.* Stroke, 2019. **50**(12): p. e344-e418.
3. Klein, M., et al., *German guidelines on community-acquired acute bacterial meningitis in adults.* Neurological Research and Practice, 2023. **5**(1).
4. Wingerchuk, D.M., et al., *International consensus diagnostic criteria for neuromyelitis optica spectrum disorders.* Neurology, 2015. **85**(2): p. 177-189.
5. Hoh, B.L., et al., *2023 Guideline for the Management of Patients With Aneurysmal Subarachnoid Hemorrhage: A Guideline From the American Heart Association/American Stroke Association.* Stroke, 2023. **54**(7).
